# Protective effect of theaflavin on glycoprotein components and TCA cycle enzymes in high-fat diet and streptozotocin-induced diabetic rats

**DOI:** 10.1186/s41936-019-0115-1

**Published:** 2019-06-21

**Authors:** Kirubananthan Gothandam, Vijayan Siva Ganesan, Thangaraj Ayyasamy, Sundaram Ramalingam

**Affiliations:** 1 Department of Biotechnology, University of Madras, Guindy Campus, Chennai, 600025 India; 2 Department of Plant Biology and Plant Biotechnology, Government Arts College for Men (Autonomous), Nandanam, University of Madras, Chennai, 600035 India; 3 Department of Medical Biochemistry, Dr. ALM Post Graduate Institute of Basic Medical Sciences, University of Madras, Taramani Campus, Chennai, 600113 India; 4 Department of Biochemistry, Saveetha Dental College & Hospital, Saveetha Institute of Medical & Technical Sciences, Velappanchavadi, Chennai, 600077 India

**Keywords:** Theaflavin, Diabetes, Glycoproteins, TCA cycle enzymes, Glucose metabolism

## Abstract

**Background:**

Theaflavins are major polyphenols in black tea which is the most widely consumed tea in the world. They possess a broad spectrum of biological activities, such as antioxidant, anti-tumor, anti-inflammatory, and cardio-protective effects. The present study was aimed to evaluate the protective effect of theaflavin on glycoprotein content and tricorboxylic acid cycle enzymes in high-fat diet and streptozotocin-induced diabetic rats as there was no study on this aspect. Diabetes was induced in male albino Wistar rats by feeding them with high-fat diet and injecting them intraperitoneally with streptozotocin (40 mg/kg b.wt).

**Results:**

Different doses of theaflavin (25, 50, and 100 mg/kg b.wt /day) were administered orally to high-fat diet and streptozotocin-induced diabetic rats for 30 days for fixing the glucose lowering dose. However, the dose at 100 mg/kg b.wt showed a significant reduction in the levels of plasma glucose and Homeostatic Model Assessment of Insulin Resistance with concomitant elevation of insulin when compared to the other two doses (25 and 50 mg/kg b.wt). Hence, 100 mg/kg b.wt was fixed as an effective dose and used for further analysis. Theaflavin administration restored the altered glycosylated hemoglobin, hemoglobin and glycoproteins (Hexose, hexosamine, fucose, and sialic acid) and TCA cycle enzymes (isocitrate dehydrogenase, α-ketoglutarate dehydrogenase, succinate dehydrogenase, and malate dehydrogenase) near the normal levels by correcting hyperglycemia. Improved histological changes were observed in the pancreas of diabetic rats upon treatment with theaflavin which supported the biochemicals investigated.

**Conclusion:**

The effect produced by the theaflavin on various parameters was comparable to that of metformin—a reference antidiabetic drug. These findings suggest that theaflavin can replace the commercial drugs which could lead to reduction in toxicity and side effect caused by the later as well as reduce the secondary completions.

## Background

Diabetes mellitus is a metabolic and endocrine disorder characterized by hyperglycemia which leads to alterations in carbohydrate, lipid, and protein metabolism, associated with absolute or relative deficiencies in insulin secretion and/or insulin action (Valiathan, 1988). More than 220 million people worldwide have diabetes, and this number is likely to more than double by 2030 (World Health and Organization (WHO), 2009).The management of diabetes with insulin and synthetic oral hypoglycemic drugs (biguanides sulfonylurea and metformin) can produce serious side effects and in addition fails to prevent diabetes-related complications in many patients. A new diabetic management strategy is needed, and it should be more effective and lessen side effects (Prabhar & Doble, 2011). In this regards, several species of plants and plant-based compounds have been described in the scientific literature as having hypoglycemic activity due to their perceived effectiveness, minimal side effects, and relatively low costs (De Sousa et al., 2004).

**Fig. 1:**
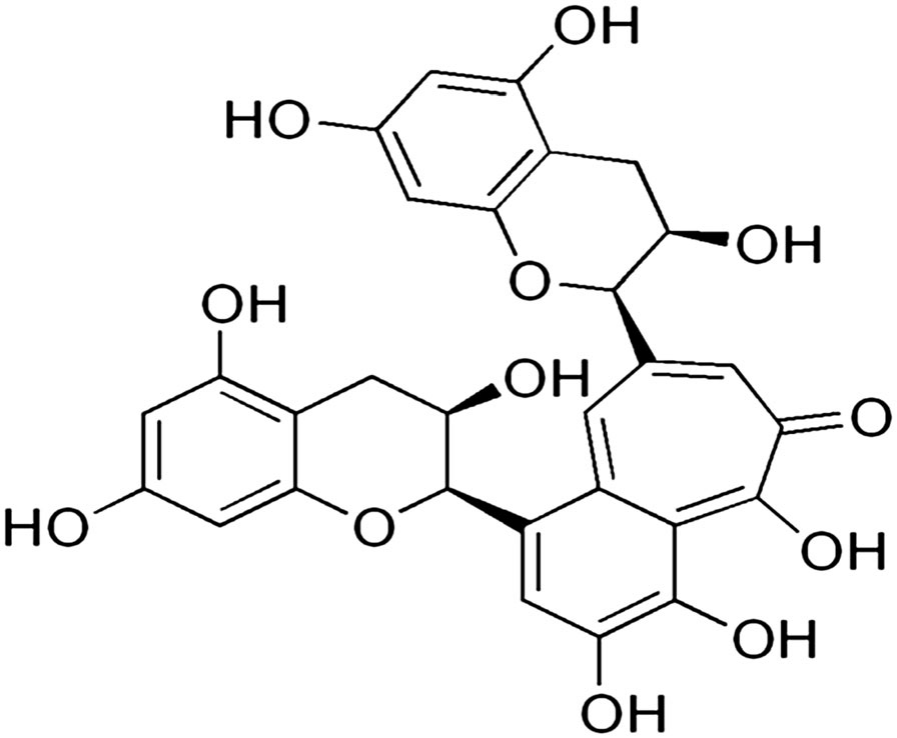
Structure of theaflavin

Theaflavins are major chemical constituents of black tea which have been reported to have antioxidant (Almajano, Carbó, Limenéz, & Gordon, 2008), anti-cancer (Gosslau et al., 2011), antiinflammatory (Zu et al., 2012), antimicrobial (Almajano et al., 2008; Friedman, 2007), and antiviral abilities including bovine coronavirus, bovine rotavirus (Clark et al., 1998), HIV-1 (Liu et al., 2005; Yang et al., 2012), influenza (Zu et al., 2012), and HSV-1 (Cantatore, Randall, Traum, & Adams, 2013). However, protective effect of theaflavin on glycoprotein components and TCA cycle enzymes in high-fat diet and streptozotocin-induced diabetic rats has not been explored so far. Therefore, the present study was aimed to evaluate effect of theaflavin on glycoprotein components and TCA cycle enzymes in diabetic rats. The structure of theaflavin was given in Fig. 1.

## Materials and methods

### Chemicals and drugs

Theaflavin and streptozotocin, high-fat diet components such as cholesterol, bile salt, egg yolk power, and lard were obtained from Sigma Chemical Company (St. Louis, MO, USA), Sisco Research Laboratories Pvt. Ltd. (Mumbai, India), Central Drug House Pvt. Ltd. (New Delhi, India), SKM Egg Products Export (India) Limited (Erode, Tamil Nadu, India), and lard was obtained from local market in Chennai. All other chemicals used were of analytical grade.

### Animal

Male albino Wistar rats weighing 200–220 g body weight were procured from the Central Animal House Facility, University of Madras, Taramani Campus, Chennai, Tamil Nadu, India. They were maintained at an ambient temperature of 25 ± 2 °C and 12/12-h of light/dark cycle. Animals were given standard commercial rat chow and water ad libitum and housed under standard environmental conditions throughout the study. The experiments were conducted according to the Institutional Animal Ethics Committee Guidelines (IAEC No 32/02/2014).

### Experimental induction of type 2 diabetes in rats

The animals were divided into seven groups of six animals each. The rats were fed with high-fat diet consisting of standard laboratory chow 84.3%, lard 5%, yolk powder 10%, cholesterol 0.2%, and bile salt 0.5% for 2 weeks (Xie et al., 2005). After 2 weeks, the animals were kept in an overnight fast and injected with low dose of streptozotocin (40 mg/kg, dissolved in 0.1 M sodium citrate buffer, pH 4.5) (Wu et al., 2012). Fasting blood glucose was measured 3 days after the injection. The rats with fasting blood glucose levels above 250 mg/dl were considered diabetic. The diabetic rats were fed on the high-fat diet for another 4 weeks.

### Experimental design

In this experiment, a total of 42 rats (30 diabetic surviving rats, 12 normal rats) were divided into seven groups with six rats in each.

- Group I: Normal control (received 0.5 ml of distilled water)
- Group II: Drug control (normal healthy control rats received intra-gastrically theaflavin 100 mg/kg b.wt.) dissolved in 0.5 ml of distilled water for 30 days.
- Group III: Diabetic control
- Group IV: Diabetic rats received intra gastrically theaflavin (25 mg/kg b.wt.) dissolved in 0.5 ml of distilled water for 30 days.
- Group V: Diabetic rats received intra gastrically theaflavin (50 mg/kg b.wt.) dissolved in 0.5 ml of distilled water for 30 days.
- Group VI: Diabetic rats received intra gastrically theaflavin (100 mg/kg b.wt.) dissolved in 0.5 ml of distilled water for 30 days.
- Group VII: Diabetic rats received intra gastrically Metformin (500 mg/kg b.wt.) dissolved in 0.5 ml of distilled water for 30 days.

### Sample collection

After 30 days of treatment, the animals were deprived of food overnight and sacrificed by decapitation. Blood was collected in two different tubes, i.e., one with a mixture of potassium oxalate and sodium fluoride (1,3) for estimation of plasma insulin, glucose, and glycoproteins and another with ethylene diamine tetra acetic acid (EDTA) for the estimation of hemoglobin and glycated hemoglobin. Liver and kidney tissues were excised immediately and rinsed in ice-chilled normal saline to remove the blood. Known weights of the tissues were minced and homogenized in 5.0 ml of 0.1 M Tris–HCl buffer (pH 7.4) in ice cold condition. The homogenate was centrifuged, and the supernatant was used for the estimation of various biochemical parameters. A section of the pancreas was kept aside for histological studies.

### Biochemical analysis

Plasma glucose was estimated by the method of Trinder using a reagent kit Trinder (1969). Hemoglobin (Hb) and glycated hemoglobin (HbA1c) were estimated by the method of Drabkin & Austin (1932) and Sudhakar and Pattabiraman (1981), respectively. The plasma insulin was measured by the method of Burgi, Briner, Franken, & Kessler (1988). Glycoprotein components such as hexose, hexosamine, fucose, and sialic acid were estimated by the methods of Dische and Shettles (1948), Dubois and Gilles (1956), Wagner (1979), and Warren (1959), respectively. The TCA cycle enzymes such as isocitrate dehydrogenase, α-ketoglutarate dehydrogenase, succinate dehydrogenase, and malate dehydrogenase were assayed by the method of Bell & Baron (1960), Mehler, Kornberg, Grisolia, & Ochoa (1948), Reed and Mukherjee (1969), and Slater & Bonner (1952), respectively. Protein content in the plasma and tissue homogenate was estimated by the method of Lowry, Rosebrough, Farr, & Randall (1951).

$$ \mathrm{HOMA}-\mathrm{IR}=\mathrm{fasting}\ \mathrm{insulin}\times \mathrm{fasting}\ \mathrm{blood}\ \mathrm{sugar}/405 $$

### Histopathology of pancreas

The pancreatic tissues of the tested rats were fixed in 10% formaldehyde, dried out in an evaluated arrangement of ethanol, and embedded in paraffin. Liver sections (5 μm thick) were acquired utilizing rotary microtome and afterward rehydrated. Sections were then stained by hematoxylin-eosin (H&E) and viewed under the light microscope and shot by photomicrography.

### Statistical analysis

All data were analyzed with SPSS/17 student software. Hypothesis testing methods included one-way analysis of variance (ANOVA) followed by LSD. All values are means of three replicates. The values are expressed as mean ± SD, and results were considered to be statistically significant if *P* value is less than 0.05.

## Results

### Dose-dependent effects of theaflavin on plasma glucose, insulin levels, and HOMA-IR index

Blood glucose and plasma insulin levels of normal and experimental rats are given in Table 1. The diabetic rats showed a significant increase in blood glucose and a significant decrease in plasma insulin levels. The administration of theaflavin (all doses) to diabetic rats caused a significant decrease in blood glucose levels and a significant increase in plasma insulin when compared with diabetic untreated rats. In the same context, diabetic rats showed a significant increase in HOMA-IR when compared with the control rats. Supplementation of theaflavin significantly decreased HOMA-IR index in a dose-dependent manner. But, theaflavin at a dose of 100 mg/kg body weight showed a highly significant effect than the other two doses (25 and 50 mg/kg b.wt) and that effect was comparable to that of metformin. Therefore, 100 mg/kg body weight was fixed as an effective dose and used for further analysis.

**Table 1:** Dose-dependent effect of theaflavin on blood glucose, insulin levels, and HOMA-IR index in control and experimental animals

Parameters	Control	Normal + theaflavin (100 mg/kg b.wt)	Diabetes	Diabetes + theaflavin (25 mg/kg b.wt)	Diabetes + theaflavin (50 mg/kg b.wt)	Diabetes + theaflavin 100 mg/kg b.wt	Diabetes + metformin (500 mg/kg b.wt)
Glucose (mg/dl)	97.65 ± 5.03	95.28 ± 5.15	273.76 ± 16.35^b^	239.43 ± 12.54^c^	183.98 ± 8.74^d^	131.81 ± 7.49^e^	129.64 ± 8.12
Insulin (μU/ml)	19.42 ± 1.80	18.99 ± 1.82	9.57 ± 1.18^b^	11.80 ± 1.12^c^	13.51 ± 1.36^d^	16.15 ± 1.35^e^	17.66 ± 1.27
HOMA-IR	4.68 ± 0.64	4.38 ± 0.53	6.62 ± 0.70^b^	7.20 ± 0.81^c^	60.2 ± 0.58^d^	5.13 ± 0.37^e^	5.57 ± 0.49

Values are given as mean ± SD for six animals in each group

Values are considered significantly different at *P* < 0.05 with post-hoc LSD test *P < 0.05

^a^Control vs. drug control (theaflavin-alone-treated rats

^b^Control rats vs. diabetic rats

^c^Diabetic rats vs. theaflavin (25 mg/kg)

^d^Diabetic rats vs. theaflavin (50 mg/kg)

^e^Diabetic rats vs. theaflavin (100 mg/kg)

^f^Diabetic rats treated with theaflavin (100 mg/kg) vs. diabetic rats treated with metformin (500 mg/kg)

### Effect of theaflavin on the levels of hemoglobin and glycosylated hemoglobin

The levels of hemoglobin and HbA1c in control and experimental animals were depicted in Table 2. The diabetic rats showed significant decrease in the level of total hemoglobin and significant increase in the levels of HbA1_C_ when compared to control rats. The levels of total hemoglobin and HbA1_C_ were significantly reversed by the administration of theaflavin and metformin to diabetic rats. Normal rats treated with theaflavin at a dose of 100 mg/kg body weight did not show any significant changes in plasma glucose, insulin, and hemoglobin and HbA1_C_ levels.

**Table 2:** Effects of theaflavin on hemoglobin and glycosylated hemoglobin levels in control and experimental animals

Parameters	Control	Normal+ theaflavin (100 mg/kg b.wt)	Diabetes	Diabetes + theaflavin (100 mg/kg b.wt)	Diabetes + metformin
Hemoglobin(g/dl)	14.62 ± 1.15	13.93 ± 0.87	7.38 ± 1.33^b^	11.96 ± 1.30^c^	12.87 ± 1.11
HbA1c(%)	4.75 ± 0.41	4.64 ± 0.42	11.15 ± 1.16^b^	7.28 ± 0.73^c^	6.40 ± 0.78

Values are given as mean ± SD for six animals in each group

Values are considered significantly different at *P* < 0.05 with post-hoc LSD test *P < 0.05

^a^Control vs. drug control (theaflavin-alone-treated rats)

^b^Control rats. vs. diabetic rats

^c^Diabetic rats.vs. theaflavin (100 mg/kg)

^d^Diabetic rats treated with theaflavin (100 mg/kg) vs. metformin (500 mg/kg)

### Effects of theaflavin on glycoprotein components

The levels of plasma and tissue glycoproteins (hexose, hexosamine, fucose, and sialic acid) are presented in Table 3. Significantly elevated levels of these glycoproteins were observed in the plasma and tissue of diabetic control rats when compared with normal rats. However, administration of theaflavin and metformin to diabetic rats for a period of 30 days resulted in a significant reduction of hexose, hexosamine, fucose, and sialic acid in plasma when compared with diabetic control rats.

**Table 3:** Effects of theaflavin on glycoprotein metabolism in control and experimental animals

Parameters	Control	Normal+ theaflavin (100 mg/kg b.wt)	Diabetes	Diabetes + theaflavin (100 mg/kg b.wt)	Diabetes + metformin (500 mg/kg)
Plasma (mg/dl)					
Hexose	86.09 ± 5.88	84.47 ± 5.80	137.56 ± 10.24^b^	91.47 ± 5.53^c^	89.35 ± 5.81
Hexosamine	70.47 ± 6.99	68.58 ± 7.03	99.34 ± 10.68^b^	80.68 ± 8.01^c^	78.59 ± 9.18
Fucose	29.43 ± 2.50	27.30 ± 2.42	42.44 ± 4.26^b^	32.39 ± 3.14^c^	30.74 ± 2.93
Sialic acid	52.26 ± 4.89	50.56 ± 4.92	73.51 ± 6.00^b^	59.64 ± 5.60^c^	58.10 ± 5.69
Liver (mg/g)					
Hexose	29.28 ± 2.61	28.59 ± 2.51	52.49 ± 5.09^b^	32.13 ± 3.21^c^	30. 77 ± 2.97
Hexosamine	13.55 ± 1.37	12. 48 ± 1.29	33.48 ± 2.95^b^	18.56 ± 1.61^c^	17.17 ± 1.46
Fucose	19.36 ± 1.84	20.43 ± 1.78	37.53 ± 3.59^b^	24.27 ± 2.47^c^	22.99 ± 2.26
Sialic acid	11.47 ± 1.04	10.81 ± 1.09	4.80 ± 0.45b	9.02 ± 0.85c	8.26 ± 0.81
Kidney (mg/g)					
Hexose	22.36 ± 2.19	21.60 ± 2.01	41.87 ± 2.89^b^	25.55 ± 2.44^c^	24.10 ± 2.22
Hexosamine	18.79 ± 1.35	17.70 ± 1.53	38.73 ± 2.32^b^	22.58 ± 1.63^c^	21.74 ± 1.59
Fucose	14.53 ± 1.34	13.53 ± 1.37	27.63 ± 2.16^b^	17.86 ± 1.66^c^	16.55 ± 1.86
Sialic acid	7.53 ± 0.61	6.85 ± 0.67	3.89 ± 0.36^b^	5.80 ± 0.56^c^	5.27 ± 0.63

Values are given as mean ± SD for six animals in each group

Values are considered significantly different at *P* < 0.05 with post-hoc LSD test *P < 0.05

^a^Control vs. drug control (theaflavin-alone-treated rats

^b^Control rats. vs. diabetic rats

^c^Diabetic rats vs. theaflavin (100 mg/kg)

^d^Diabetic rats treated with theaflavin 100 mg/kg vs. metformin (500 mg/kg

The levels of sialic acid were significantly decreased whereas the levels of hexose, hexosamine, and fucose were significantly increased in the tissues of diabetic rats. Administration of theaflavin and metformin to diabetic rats significantly reversed these changes in tissues to near normal levels.

### Effects of theaflavin on TCA cycle key enzymes

Table 4 represent the activities of TCA cycle key enzymes in liver and kidney tissues of control and experimental rats. The liver and kidney tissues of diabetic rats showed a significant decrease in the activities of isocitrate dehydrogenase, α-ketoglutarate dehydrogenase, succinate dehydrogenase, and malate dehydrogenase. The diminished activities of these key enzymes were reverted to close normalcy by treatment with theaflavin and metformin to diabetic rats.

**Table 4:** Effects of theaflavin on TCA cycle enzymes in control and experimental animals

Parameters	Control	Normal+ theaflavin (100 mg/kg b.wt)	Diabetes	Diabetes + theaflavin (100 mg/kg b.wt)	Diabetes + metformin (500 mg/kg b.wt)
Liver					
Isocitrate dehydrogenase	682.75 ± 45.47	680.26 ± 45.57	371.44 ± 36.53^b^	612.37 ± 40.45^c^	610.66 ± 40.19
Malate dehydrogenase	393.23 ± 40.16	389.99 ± 40.21	171.17 ± 22.40^b^	304.09 ± 27.48^c^	302.34 ± 27.67
Alpha ketoglutarate dehydrogenase	284.35 ± 30.35	282.68 ± 20.76	104.5 ± 11.72^b^	200.28 ± 15.87^c^	198.37 ± 15.88
Succinate dehydrogenase	56.39 ± 4.85	54.09 ± 5.20	14.60 ± 2.66^b^	36.16 ± 3.21^c^	34.51 ± 2.86
Kidney					
Isocitrate dehydrogenase	615.41 ± 24.55	612.79 ± 24.47	297.21 ± 17.51^b^	591.13 ± 22.11^c^	589.39 ± 22.02
Malate dehydrogenase	323.44 ± 23.62	322.61 ± 22.90	116.51 ± 13.23^b^	283.32 ± 19.89^c^	281.56 ± 19.96
Alpha ketoglutarate dehydrogenase	85.05 ± 6.39	82.25 ± 6.62	24.45 ± 3.61^b^	68.54 ± 6.06^c^	66.85 ± 6.05
Succinate dehydrogenase	30.31 ± 2.70	28.29 ± 2.65	13.31 ± 1.18^b^	22.94 ± 2.44^c^	21.07 ± 2.43

Values are given as mean ± SD for six animals in each group

Values are considered significantly different at *P* < 0.05 with post-hoc LSD test *P < 0.05

The enzyme activities are expressed as: *n* moles of a-ketoglutarate/h/mg of protein for isocitrate dehydrogenase, *n* moles of potassium ferrocyanide/h/mg of protein for α-ketoglutarate dehydrogenase, μmoles of succinate oxidized/min/mg of protein for succinate dehydrogenase, and μmoles of NADH oxidized/min/mg of protein for malate dehydrogenase

^a^Control vs. drug control (theaflavin-alone-treated rats

^b^Control rats vs. diabetic rats

^c^Diabetic rats vs. theaflavin (100 mg/kg)

^d^Diabetic rats treated with theaflavin (100 mg/kg) vs. metformin (500 mg/kg)

### Histopathological observations of pancreas

**Fig. 2:**
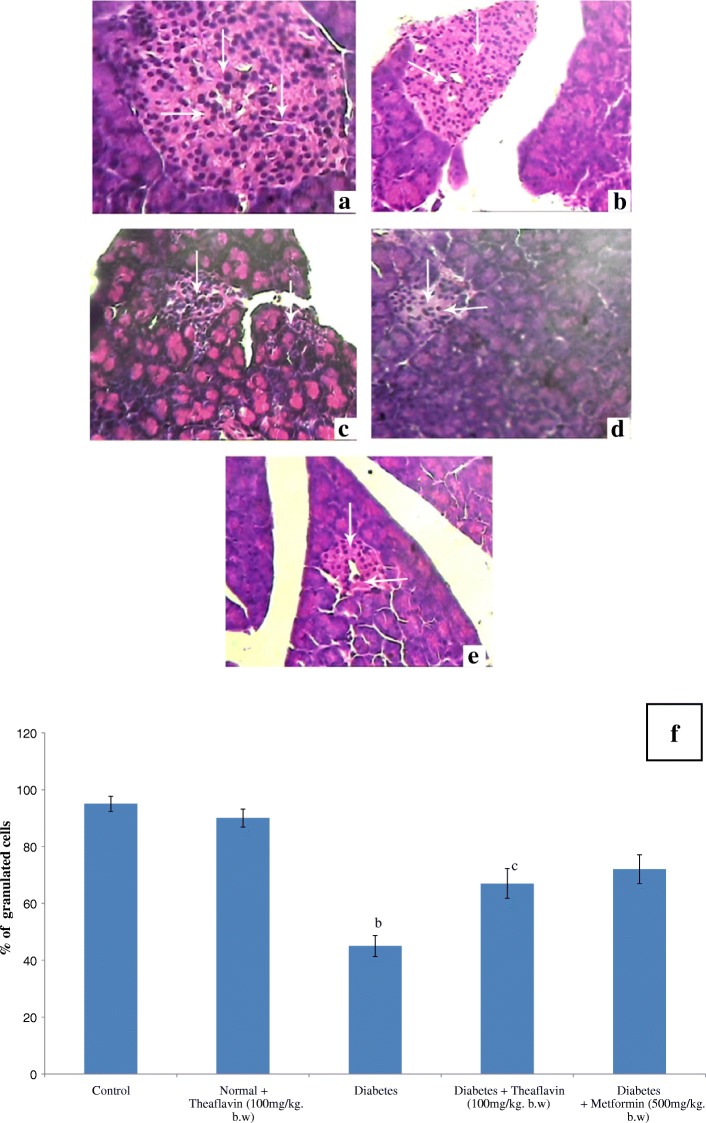
Histopathological section of pancreas of control and experimental rats (× 40). Control (**a**), normal + theaflavin (**b**), diabetes induced (**c**), diabetic + theaflavin (**d**), and diabetic + metformin (**e**). **f** Quantification of granulated cells from pancreas. Values are given as mean ± SD for six animals in each group (*n* = 6).Values are considered significantly different at *P* < 0.05 with post hoc LSD test. Comparisons are made as (a) control vs. drug control (theaflavin-alone-treated rat); (b) control rat vs. diabetic rat; (c) diabetic rat vs. theaflavin-treated diabetic rat; (d) theaflavin-treated diabetic rat vs. metformin

Figure 2 represents the hematoxylin and eosin staining of pancreas of control and experimental rats. Normal histological features of both exocrine and endocrine part were shown in the section of pancreatic tissue of control rats (Fig. 2a). Pancreatic section from theaflavin-alone treated rat shows no changes on beta cells, and it looks like control pancreas (Fig. 2b). The section of pancreatic tissues of diabetic rats shows degenerative changes of islets and is characterized by reduction in number and size of the islets. Subsequently, the central areas of most pancreatic islets are completely empty when compared to control rats (Fig. 2c). The section of pancreatic tissues of diabetic rats treated with theaflavin and metformin shows marked regeneration of islets with significant number of granulated cells than that of diabetic rats. Moreover, several islets are well granulated when compared to pancreatic islets of diabetic rats indicating the improved functioning of cells which was reflected in the increased plasma insulin level (Fig. 2d, e).

## Discussion

Metabolic disorder of glucose is the most important and fundamental pathological changes in diabetes. So, the blood glucose level is the key indicator to evaluate the success of models and the effectiveness of drugs. Experimental results showed that the drugs can significantly reduce high blood sugar, regulate the glycogen synthesis which was very significant to maintain normal blood sugar and improve glucose tolerance. Hence, blood glucose is a key marker for diagnosis and prognosis of diabetes mellitus. Insulin deficiency causes radical elevation in the levels of blood glucose as a result of excessive production of endogenous glucose by hepatic as well as extrahepatic tissues through gluconeogenic and glycogenolytic pathways and reduced consumption of glucose through glycolytic, TCA cycle, glycogenic, and HMP shunt pathways by various tissues during diabetes mellitus (Soling & Kleineke, 1976). Moreover, Insulin deficiency and high levels of plasma glucose in diabetic condition may result in an increased synthesis of oligosaccharide moieties of glycoprotein; hexose, hexosamine, fucose, and sialic acid have an important role in the maintenance of structural integrity of the membrane bilayer. Cell surface glycoproteins have vital roles in the transport of vitamins and lipids, in signal transduction as hormone receptors and in immunological specificity.

In the present study, upon treatment with theaflavin and metformin appreciably lowered the level of blood glucose and improved the insulin in high-fat diet and streptozotocin-induced diabetic rats. These glucose lowering and elevated insulin levels are brought by the antioxidant potential of theaflavin (Almajano et al., 2008). The consumption of natural antioxidant phytochemicals were reported to have potential health benefits and help to regenerate β cells and protect pancreatic islets against cytotoxic effects of streptozotocin (Alvarez et al., 2004; Rangkadilok et al., 2007).

Glycosylated hemoglobin (HbA1c) is the clinical marker of chronic glycemic control in patients with diabetes mellitus (Koenig et al., 1976). Persistent hyperglycemia leads to the glycosylation of amino groups of lysine residue in proteins (Asgary, Naderi, & Sarrafzadegan, 1999). This condition favors reduction in the level of total hemoglobin and elevation in glycosylated hemoglobin which in turn directly proportional to blood glucose (Al-Yassin & Ibrahim, 1981). Diabetic rats showed higher levels of glycosylated hemoglobin indicating their poor glycemic control. The oral administration of theaflavin and metformin to diabetic rats significantly reduced the HbA1c levels compared to untreated diabetic rats. This reflects the antioxidant potential theaflavin and metformin in long-term control of hyperglycemia through insulin secretion.

The function of glycoproteins in stabilizing the tissue may be involved in maintaining the structural stability of collagen fibrils. Glycoproteins are important components of intracellular matrix, cell membrane, and membranes of sub-cellular organelles (Zachariah & Basu, 1993). During diabetes, synthesis of glycoproteins was decreased because of reduced incorporation of glucose caused by insulin deficiency. Increased glycosylation of various proteins in diabetic patients have also been reported earlier and the elevated of glycoproteins in diabetics may also be a predictor of angiopathic complications (Konukoglu, Serin, Akcay, & Hatemi, 1999; Rahman, Zafar, & Shera, 1990) In this study, we have observed the altered levels of hexose, hexosamine, fucose, and sialic acid in plasma and tissues of streptozotocin and high-fat diet-induced diabetic rats. Theaflavin and metformin administration to diabetic rats normalized the levels of glycoproteins in plasma and tissues. Decreased hyperglycemic state with increased levels of plasma insulin observed in theaflavin- and metformin-treated diabetic rats might be responsible for the beneficial changes in glycoproteins in the plasma, liver, and kidney. In this context, other researchers have shown that a decrease in hyperglycemia could lead to a decrease in glycoprotein levels (Gandhi & Chowdhury, 1979; Latha & Pari, 2005; Sundaram, Naresh, Shanthi, & Sachdanandam, 2012).

Mitochondria play a part in regulation of diverse physiological functions by providing energy for the majority of intracellular processes necessary for vital functions. Mitochondria are one of the key cell organelle in diabetes research because of their central role as a regulator of energy balance (Wallace, 1999). Magnetic resonance spectroscopy studies on human suggest that more subtle defects in mitochondrial function might take part in the pathogenesis of insulin resistance in diabetes which is the most widespread metabolic disease in the world (Lowell & Shulman, 2005). In the present study, the activities of the mitochondrial marker enzymes—isocitrate dehydrogenase, α-ketoglutarate dehydrogenase, succinate dehydrogenase, and malate dehydrogenase levels—were significantly dropped in the liver and kidney of high-fat diet and streptozotocin-induced diabetic rats. These results are in agreement with previous reports (Akude et al., 2011; Sener, Rasschaert, & Malaisse, 1990). However, diabetic rats treated with theaflavin and metformin resulted in significant marked elevation in the activities of TCA cycle enzymes which might be due to the normoglycemic potential of theaflavin and metformin.

The population of β cells in islets of Langerhans of the pancreas reflects the production and secretion of insulin. In the present study, histopathological findings revealed severe necrotic changes of pancreatic islets and a relatively small number of insulin-positive β cells were observed in high-fat diet and streptozotocin-induced diabetic rats with such evidence, it is possible to assume that theaflavin treatment might have stimulated the insulin secretion from existing β cells.

## Conclusion

Based on the results obtained, we conclude that oral administration of theaflavin to diabetic rats restored all the altered glycoprotein components and TCA cycle enzymes to near normal levels by correcting the hyperglycemia. These findings suggest that theaflavin has complimentary potency to develop an antihyperglycemic agent for the treatment of diabetes mellitus. Further studies are in progress to elicit the exact mechanism of antihyperglycemic action of theaflavin in diabetes.

## Abbreviations

Hb-hemoglobin: HbA1c-Glycosylated hemoglobin
TCA cycle: Tricorboxylic acid cycle
WHO: World Health Organization
